# How Difficult Is It to Fold a Knotted Protein? *In Silico* Insights from Surface-Tethered Folding Experiments

**DOI:** 10.1371/journal.pone.0052343

**Published:** 2012-12-20

**Authors:** Miguel A. Soler, Patrícia F. N. Faísca

**Affiliations:** 1 Centro de Física da Matéria Condensada, Universidade de Lisboa, Lisboa, Portugal; 2 Departamento de Física, Universidade de Lisboa, Lisboa, Portugal; University of Michigan, United States of America

## Abstract

We explore the effect of surface tethering on the folding process of a lattice protein that contains a trefoil knot in its native structure via Monte Carlo simulations. We show that the outcome of the tethering experiment depends critically on which terminus is used to link the protein to a chemically inert plane. In particular, if surface tethering occurs at the bead that is closer to the knotted core the folding rate becomes exceedingly slow and the protein is not able to find the native structure in all the attempted folding trajectories. Such low folding efficiency is also apparent from the analysis of the probability of knot formation, *p_knot_*, as a function of nativeness. Indeed, *p_knot_* increases abruptly from ∼0 to ∼1 only when the protein has more than 80% of its native contacts formed, showing that a highly compact conformation must undergo substantial structural re-arrangement in order to get effectively knotted. When the protein is surface tethered by the bead that is placed more far away from the knotted core *p_knot_* is higher than in the other folding setups (including folding in the bulk), especially if conformations are highly native-like. These results show that the mobility of the terminus closest to the knotted core is critical for successful folding of trefoil proteins, which, in turn, highlights the importance of a knotting mechanism that is based on a threading movement of this terminus through a knotting loop. The results reported here predict that if this movement is blocked, knotting occurs via an alternative mechanism, the so-called spindle mechanism, which is prone to misfolding. Our simulations show that in the three considered folding setups the formation of the knot is typically a late event in the folding process. We discuss the implications of our findings for co-translational folding of knotted trefoils.

## Introduction

Eighteen years have passed since the discovery of the first knotted protein, the human carbonic anhydrase B (2cab.pdb) [1]. A very recent survey of the Protein Data Bank (PDB) revealed the existence of 398 knotted-proteins and 222 proteins whose native structure contains a slipknot [2]. The most common knot in the PDB is the trefoil (also known as the 31) knot [2], [3], and the most topologically complex protein found so far is protein DhHI (3bjx.pdb) whose backbone is tangled in a Stevedore (or 61) knot [4].

Knotted proteins stand for as extreme examples of topological complexity and despite forming a small subset of the PDB (knotted and slip-knotted proteins account for less than 1% of all available protein structures in the PDB), their very existence triggered several lines of research oriented to understand the functional role of knots in proteins, their effect on protein stability, and the way they fold [5]. It has been suggested that the process of knotting, clearly an additional complication to the already challenging folding mechanism, could be compensated by some added functional advantage of knotted proteins over their unknotted counterparts [3].

In this view, the analysis of specific knotted proteins suggested a role against unfolding and degradation by the proteasome in protein human ubiquitin hydrolase (1xd3.pdb) [3], and enhancement of thermal and mechanical stability when the knot is located deeply within the protein sequence as in protein human ornithine transcarbamylase (1yh1.pdb) [6], or in an engineered form of carbonic anhydrase II [7]. Additionally, a comprehensive analysis that considered *all* the knotted and slip-knotted proteins in the PDB concluded that slipknots may be important stabilizing elements in transmembrane transporting channels [2]. Slipknots were firstly identified by King and Yeates who noted their unusual occurrence in certain transmembrane proteins [8].

With a few exceptions [4], [9], research on the folding mechanism of knotted proteins has been focusing on trefoil knots. A seminal computational study by Wallin and co-workers, based on a coarse-grained C-alpha model and Langevin dynamics, studied protein YiBK (1j85.pdb), a methyltransferase that contains a deep trefoil knot at approximately 40 amino acids from the C-terminus [10]. It was shown that specific non-native interactions are necessary to achieve successful folding by mediating the threading of the C-terminus through a knotting loop formed by the preceding chain segment. Although knotting could be observed in conformations with a small number of native contacts it occurred with highest probability in highly native-like conformations. A functional role for non-native interactions in the folding of trefoil proteins was also reported in a recent contribution based on Monte Carlo (MC) simulations of a C-alpha protein representation [11]. This study, which focused on knotting events occurring early on in the folding process, showed that threading the C-terminus through loops formed in a loosely formed protein globule is facilitated by establishing non-native stabilizing interactions between the C-terminus and other parts of the chain.

While the importance of non-native interactions in the folding energetics of knotted proteins appears to be important, and actually determinant for early knot formation [11], it is also known that non-native interactions are not strictly necessary to tangle the polypeptide chain. A study by Sułkowska and Onuchic, based on a C-alpha protein representation and Langevin Dynamics, showed that if protein energetics is exclusively driven by native interactions then the most probable knotting mechanism in YibK involves an intermediate conformation with a slipknot forming with highest probability toward late folding [12]. In the slipknot conformation the terminal part of the polypeptide chain forms a hairpin (which acts like a ‘hook’) that threads a native-like loop formed by another region of the polypeptide chain. A recent study by the same group, which investigated the smallest protein in the PDB with a trefoil knot (2efv.pdb), used a full atomistic protein representation combined with Molecular Dynamics (MD) simulations, to reveal the importance of a folding mechanism based on the late formation of slipknots [13].

On the experimental front Jackson and co-workers have attained considerable insight into the folding mechanism of knotted proteins from *in vitro* studies on YibK and YbeA (1o6d.pdb) [14]. A general conclusion from equilibrium and kinetic measurements is that these knotted proteins have complex energy landscapes, and fold through parallel pathways that are populated by several intermediate states, some of them highly native-like [15]. Ingenious experiments developed by the same group, which explored the folding kinetics of specific structural constructs (circularized [16] and fusion [17] forms) of proteins YikK and YbaA revealed the somewhat surprising result that they are able to keep their knotted topology even under strongly denaturing conditions. The resilience of the denatured state to remain knotted has been pointed out as the reason why these proteins are able to fold efficiently *in vitro*.

Recent experiments that started to shed light on the folding mechanism of knotted proteins in the cellular environment revealed that knotting is the rate limiting step in the folding process of newly synthesized (and therefore unknotted) chains [18]. This discovery is in line with previous studies [19], including one report based on phi-value analysis [20]. Although unknotted chains can fold spontaneously and efficiently (in the sense that they do not populate misfolded intermediates) their folding rate is small but significantly enhanced by chaperonins, suggesting an important role for these control systems in folding knotted proteins *in vivo* [18].

In this work we explore the effect of surface tethering on the folding process of a lattice protein (i.e. a coarse grained model that reduces the protein backbone to a string of single beads placed on the vertices of a cubic lattice) that was designed to contain a trefoil knot in its native structure via extensive MC simulations. This model system was previously introduced in Ref. [21], which focused on the study of its bulk folding properties. Surface-tethering is relevant not only in the cellular context, where folding nascent chains are bound to the ribosome by their C-terminus during protein synthesis, but also in the context of single-molecule experiments where the investigated protein is sometimes linked by one of its termini to a chemically inert surface. The major goal of this study is to investigate the dependence of knotting efficiency on conformational and steric constraints imposed upon the tethered chain in order to shed light on the folding mechanism of proteins with trefoil knots (e.g., is knotting facilitated or impaired by preventing the movement of one of the protein termini?). The reverse question has been recently addressed in Ref. [22], where it was shown via MD simulations that the way in which a knotted protein is untied depends critically on which part of the protein is pulled (just like a shoelace is more easily untied if one pulls the ends rather than the loops).

The use of lattice models and MC simulations has a long tradition in the study of protein folding [23], [24], [25], [26], [27], [28], [29], [30], [31], [32], [33], [34], [35], and, more recently, in exploring the related problems of co-translational folding [36], protein aggregation [37] and binding [38], just to mention a few examples. A main advantage of these minimalistic models over alternative off-lattice representations is their computational efficiency. Indeed, since lattice simulations run in a relatively short amount of computer time, it is possible to generate very large statistical samples of the whole folding process, and observe many transitions between the denatured and native states, which allow an accurate evaluation of folding thermodynamics and kinetics. In this study we make use of this advantage and perform an in-depth comparative study of the folding thermodynamics, kinetics and knotting mechanism of a knotted trefoil protein in different folding setups: folding freely in the bulk, and folding tethered to a chemically inert plane by each one of its terminus. Apart from allowing for an exploration of the effects of surface tethering in the folding process, which is relevant for single-molecule experiments, the adopted approach also provides insight into the knotting mechanism.

## Methods

### The Simple Lattice Gō Model and Monte Carlo Folding Simulation

We consider a simple cubic lattice model of a protein molecule with chain length *N.* In the simple lattice representation the protein is reduced to its backbone structure: amino acids, modeled by beads of uniform size, are placed on the vertices of a regular three-dimensional lattice and the peptide bond, which covalently links amino acids along the polypeptide chain, is represented by sticks with uniform (unit) length corresponding to the lattice spacing. The latter represents the distance between two C_α_ atoms along the polypeptide chain. In order to satisfy the excluded volume constraint only one bead is allowed per lattice site.

To model protein energetics we use the Gō potential [39]. In the Gō potential the energy of a conformation, defined by the set of bead coordinates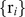, is given by the contact Hamiltonian.
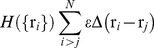
(1)where *ε* is the (uniform) interaction energy parameter (generally taken as −1) and the contact function 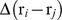, is unity only if beads *i* and *j* form a native contact and is zero otherwise.

In order to mimic the protein's relaxation towards the native state we use the Metropolis Monte Carlo (MC) algorithm [40] together with a local move set that includes corner-flips and end-moves, which displace one bead at a time (the end-moves are exclusively used to move the chain’s termini and the corner-flip is used to displace all the other beads in the chain), and the crankshaft move (which involves the simultaneous displacement of two beads except termini beads). The adequacy of the adopted move set to study polymer dynamics including the special case of knotted polymers was established in Ref. [41]. A MC simulation starts from a randomly generated unfolded conformation and folding progress is monitored through several properties (e.g., the fraction of the established native contacts, *Q*, and the gyration radius, *R_g_*). Further details on the adopted simulation algorithm can be found elsewhere [25], [26], [42].

Except otherwise stated all the results presented in this work were evaluated at the melting temperature *T_m_*. The melting temperature is determined by the co-existence of the lowest energy structure, the native structure, and a multitude of high-energy ones [43]. It is generally estimated from differential scanning calorimetry experiments as being the temperature at which the heat capacity attains its maximum value. For a strict two-state transition (i.e. a folding transition which does not populate intermediate states), *T_m_* can thus be defined as the temperature at which denatured and native states are equally populated at equilibrium. Throughout the paper the temperature is measured in units of *ε*/k_B_, where k_B_ is the Boltzmann constant.

### Folding Thermodynamics

In order to explore the thermodynamics of the folding transition and compute equilibrium properties we have conducted very long (10^9^ MC-steps per residue) replica-exchange (RE) MC simulations at 40 different temperatures. Each MC trajectory consists of - at least –10^6^ MCS per residue after equilibration. The temperature grid for the RE has been established to ensure a 100% acceptance ratio for the RE-move attempts. In the course of a single simulation the replica reliably and repeatedly visits all the temperatures in the grid with cycle time of 40 replica exchange moves, while a single total simulation comprised at least 25 full cycles. This indicates good convergence quality of our simulation data. The results reported here correspond to an average of three RE simulations.

The heat capacity *C_v_* is evaluated from the mean squared fluctuations in energy at each temperature considered in the RE simulations according to the definition 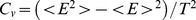. The free energy as a function of selected reaction coordinates was evaluated with the weighted histogram analysis (WHAM) method [44].

### Folding Kinetics

To obtain kinetic properties such as the folding rate, we have carried out fixed temperature MC simulations at *T_m_*. To get statistically significant kinetic measurements, we recorded 2000 independent MC folding runs. The corresponding folding times (i.e. first passage times) allow evaluating the distribution of proteins which remain unfolded as a function of MC ‘time’ (i.e. MC steps). The folding rate is given by the slope of the linear fitting of this distribution to a single-exponential decay.

### Knot Detection

The native structure of the knotted protein studied in this work contains a trefoil knot. In order to determine if, whether or not, a conformation sampled in the course of the folding simulation is knotted we used an adapted version of the Koniaris-Muthakumar-Taylor (KMT) algorithm [45], which reduces the lattice backbone to the smallest segment that contains the knot. Details on the adopted procedure can be found elsewhere [21].

### Structural Clustering

In order to determine the relevant conformational classes present in an ensemble of conformations we have used the hierarchical clustering algorithm *jclust* available in the MMTSB tool set [46]. Since we are using a lattice model, clustering is done based on contact map similarity. We set the maximum of clusters to 8. From each identified cluster we extract the conformation that is the closest to the cluster’s centroid.

### Statistical Error

The statistical error in the measurements reported in the Results section is not shown because it is of the same size or smaller than that of the adopted symbols.

## Results and Discussion

### Model Systems

**Figure 1:**
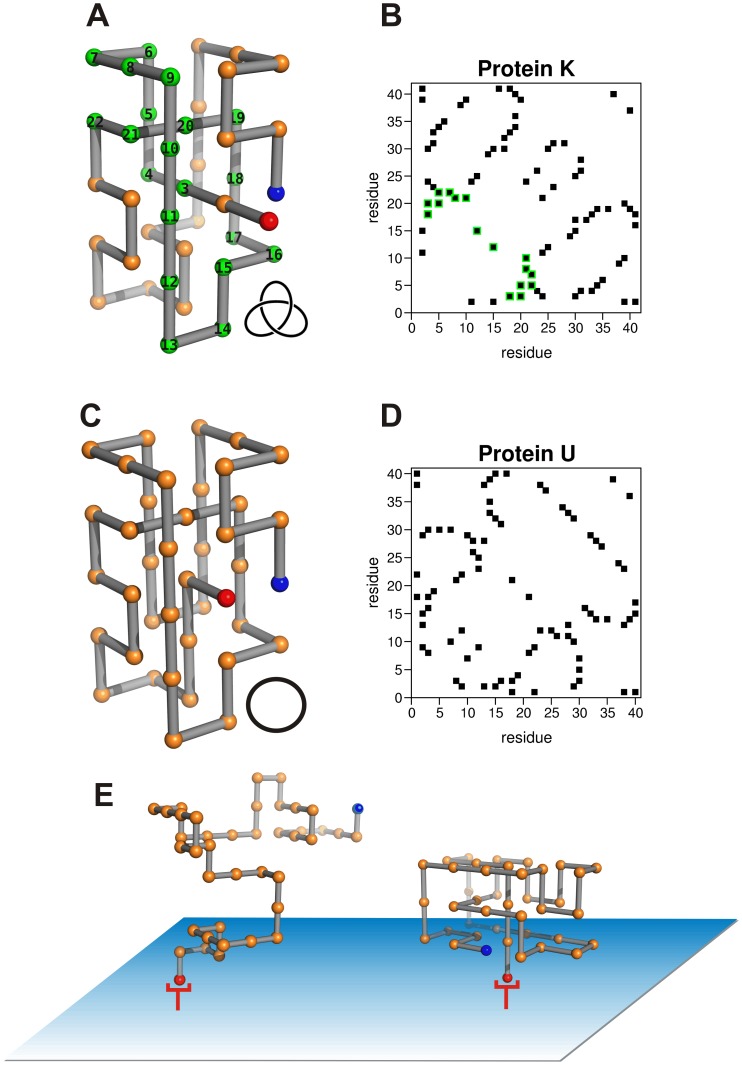
The native structure of the lattice trefoil (protein K) explored in this study. In panel (A) we show the three-dimensional representation and in (B) the contact map representation. In (A) the minimal chain segment that contains the knot, extending from bead 3 to bead 22, is highlighted. Bead 1, representing the C-terminus, is colored red and the N-terminus is colored blue. In (B) the 8 native contacts that are established between the residues that make up the knotted core is highlighted. Panels (C) and (D) represent the native structure and contact map of the unknotted lattice protein. In (E) we illustrate the folding setup where the C-terminus is linked to an inert plane by a linker whose size is one-lattice spacing. The trefoil symbol is used to indicate that conformation K is knotted and the circle is used to indicate that conformation U is unknotted.

In this work we focus on a lattice protein, termed protein K, which was designed to have its backbone arranged in the form of a trefoil knot. A detailed description of this model system can be found in Ref. [21]. Here we summarize its main features. The native structure of protein K is represented in Figure 1A, where the minimal chain segment that contains the knot (the so-called knotted core) has been highlighted. The contact map of Figure 1B shows the 40 contacts present in the native structure, 8 of which are established between the beads that make up the knotted core. The knotted core is located 3-beads away from the bead colored in red (that we numbered by convention bead 1), and 20-beads away from the bead colored blue (bead number 41). The proximity of the knotted core to one of the termini is observed in real proteins with trefoil knots. Indeed, we have noticed that the knotted core is located between 1 and 5 residues away from the C-terminus in the vast majority (74%) of the knotted trefoils reported in the pKNOT web server [47]. Interestingly, in the remaining (few) cases, where the knotted core sits more deeply inside the protein sequence, it stays also closer to the C-terminus. Thus, our lattice system captures a common structural property of knotted trefoils, i.e., the preference of the knotted core to be closer to the C-terminus. More specifically, our model system can be taken as a coarse grained representation for protein carbonic anhydrase MTH1 (1k3r.pdb) from *Methanothermobacter thermautotrophicus* (see, e.g., Table 1 from Ref. [3]) in the sense that in both structures the knotted core represents 7.3% of the total chain length.

Figure 1C shows the native structure of protein U, which is unknotted and will be used as a control system in some of the experiments reported here. This target structure was built from protein K by suitably tweaking its backbone in order to remove the knot. As a result there is a very high overlap of 90% between the two structures (only four backbone segments do not coincide) when they are optimally superimposed. Figure 1D shows the 40 native contacts of the unknotted fold. The chain length of the knotted fold (*N* = 41) is one unit larger than that of the unknotted one so that the extension of the first terminus above the cuboid’s surface guarantees that both termini can be connected unambiguously to form a closed loop (i.e. to form a topological knot).

### Folding Setup

We study the folding process of each model system when the chain is connected to a chemically inert planar surface with a linker of the size of one lattice spacing (Figure 1E). Since the plane is chemically inert, the only existent interactions between the protein and the plane are steric interactions (excluded volume interactions). In this study tethering occurs either at the first or at the last bead. By analogy with real trefoil proteins we term the bead that is closest to the knotted core as the C-terminus and the other bead will be the N-terminus. Blocking the movement of the C-terminus upon surface tethering will allow evaluating the importance of a knotting mechanism that is based on a threading movement of the C-terminus and to disclose alternative knotting mechanisms that may come into play upon surface tethering.

Apart from disrupting the translational movement of the chain, tethering eliminates the possibility to ‘end-move’ the bead that is linked to the surface. This geometrical constraint has a very simple interpretation in the context of the adopted MC simulation framework: it is effectively equivalent to change the move set by removing the end-moves that displace one of the chain’s termini. Furthermore, the presence of a nearby plane has the additional effect of effectively reducing the folding conformational space. The outcome of the surface tethered folding experiment will depend on the size of the linker. Here, we restrict ourselves to a linker with the size of one lattice spacing in order to enhance as much as possible the effects of the nearby surface on the folding process. For control reasons we also investigate folding in the bulk (which mimics standard *in vitro* folding).

### Folding Thermodynamics

**Figure 2:**
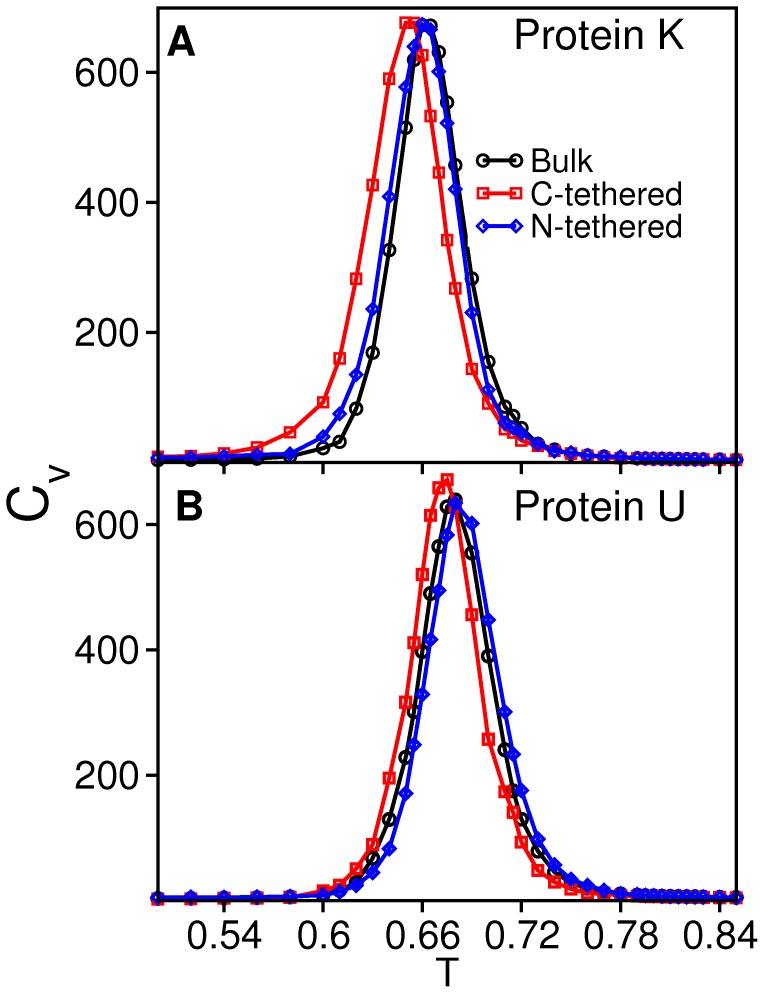
The heat capacity as a function of temperature in the considered folding setups. Panel (A) refers to the knotted protein and panel (B) to the unknotted one. The melting temperature *T_m_* is the temperature at which the heat capacity attains its maximum value.

The results reported in Figure 2 for the variation of the heat capacity with temperature show robustness of the melting temperature *T_m_* upon surface tethering for both the knotted and unknotted proteins. Indeed, there is only a negligible decrease of *T_m_* upon linking the C-terminus to the plane.

**Figure 3:**
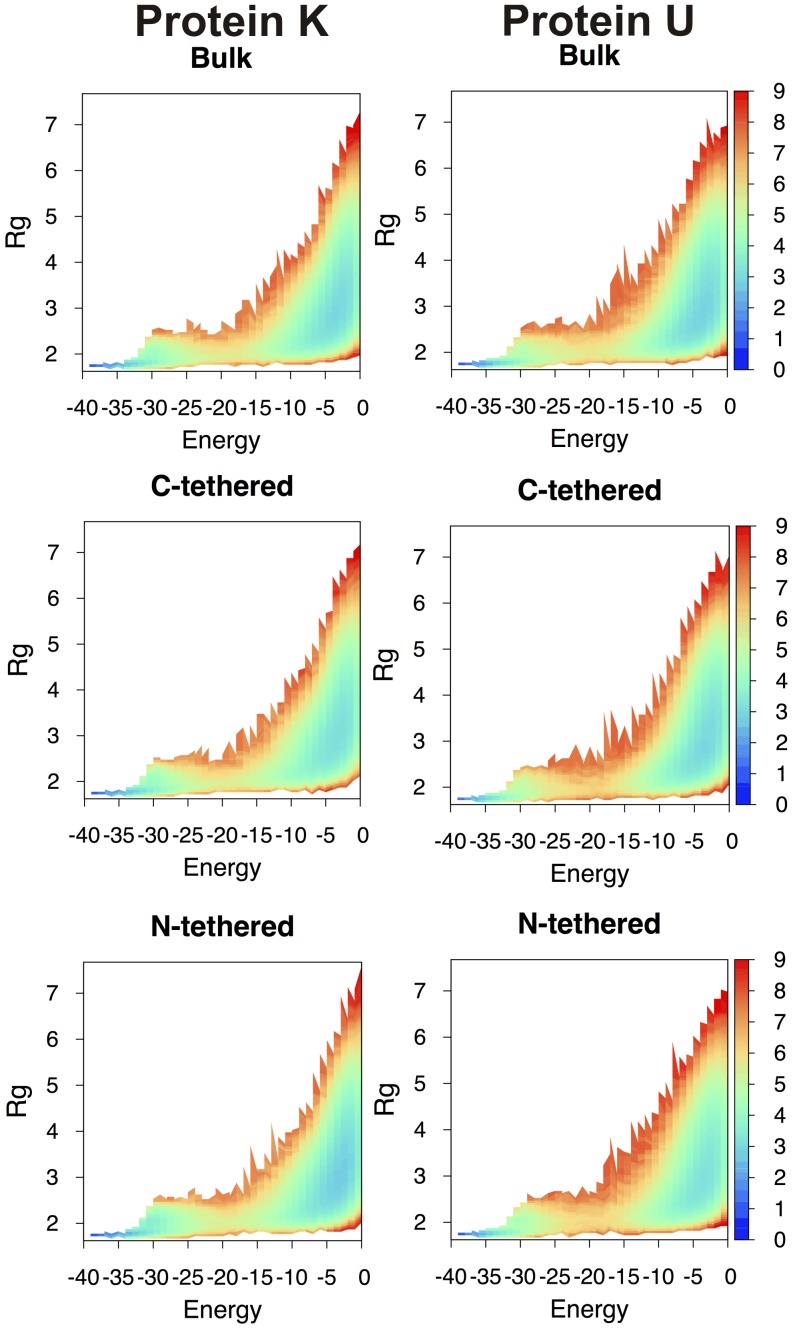
Free energy surfaces at *T_m_*. The free energy as a function of energy (which is equivalent to minus the number of established native contacts when protein energetics is modeled by the Gō potential) and gyration radius, *R_g_* (measured in angstroms), evaluated at *T_m_* for the knotted (left) and the unknotted protein (right) in the considered folding setups. Since the native state is unequivocally defined by a unique conformation it appears as one point in the free energy surface (instead of being represented by a native basin as in the case of off-lattice models).

The analysis of the free energy surfaces (Figure 3) indicates that the existence of a knot in the native structure leads to a slight stabilization of the transition state (TS) region relative to the unknotted protein. This observation is in line with results reported in Ref. [10], although in that case the stabilization of the TS was ascribed to non-native interactions.

**Figure 4:**
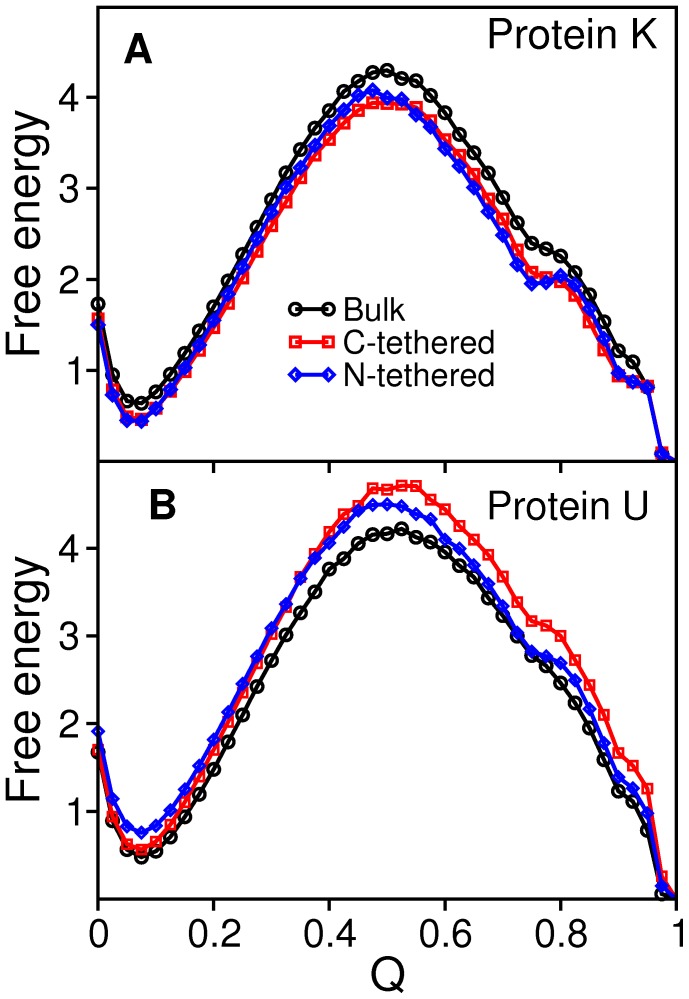
Free energy profiles. The free energy as a function of the fraction of native contacts for the knotted protein (A) and the unknotted one (B) evaluated at *T_m_.*

The one-dimensional projections of the free energy (Figure 4) reveal an important effect of surface tethering (either at the N- or at the C-terminus) upon the folding process, which is particularly pronounced for protein K: the development of a post-TS intermediate basin whose minima is located at *Q* = 0.75. This basin is incipient in the free energy curve corresponding to the bulk setup, suggesting that excluded volume interactions with the nearby plane enhance the intrinsic propensity of the knotted fold to populate intermediate states. In the free energy profiles of the unknotted protein this feature appears in a vestigial form. This should not be taken as surprising because the unknotted structure was built directly from the knotted one. Therefore, it retains its gross topological features, which are imprinted in the observed folding behavior.

In what follows we measure the folding kinetics of the knotted protein, and explore the conformational traits of the intermediate state located at *Q* = 0.75 in the surface tethered setups.

### Folding Kinetics

**Figure 5:**
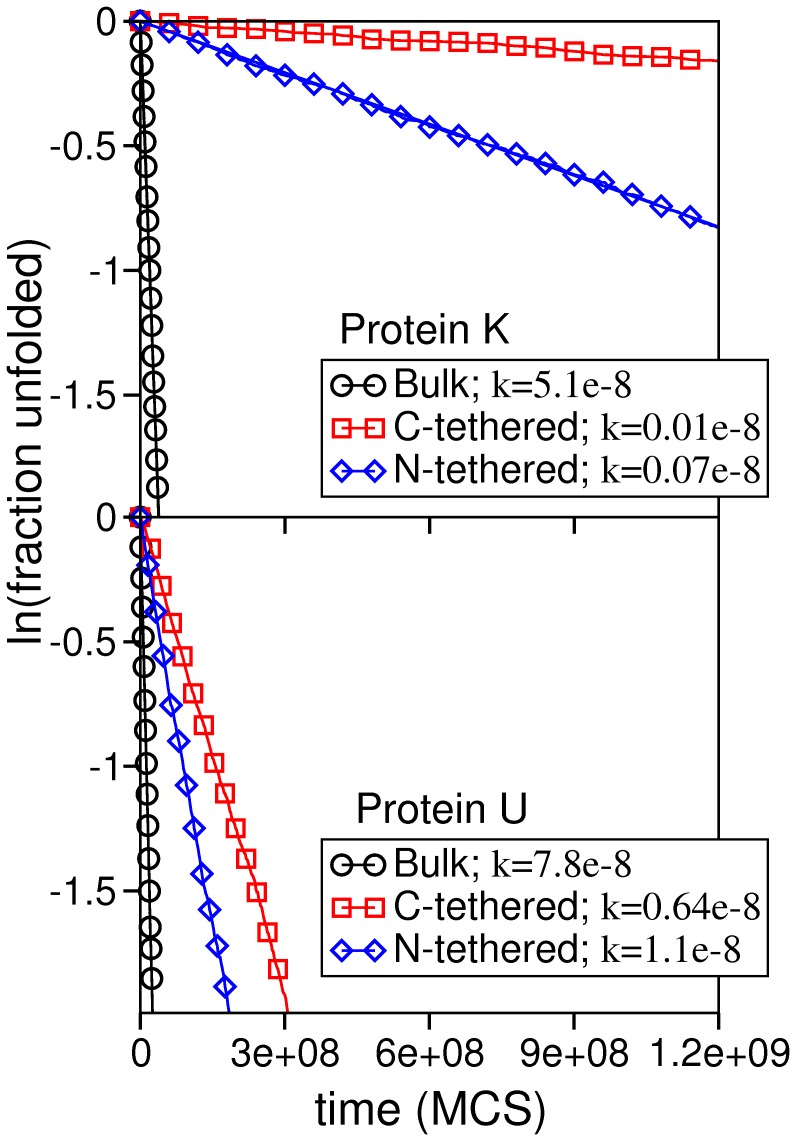
The folding rate for protein K and protein U in the considered folding setups evaluated at *T_m_*.

We have determined the folding rate of the knotted (and unknotted) protein in the different tethering setups and we report our findings in Figure 5, where the folding rate observed in the bulk is also shown. We start by mentioning that the folding rate of the surface-tethered knotted protein is an estimated quantity. Indeed, the protein was not able to find the native structure in all the attempted folding runs (each run consisting of a total number of 4×10^9^ MCS). In other words, ‘foldicity’ [48], defined as the ratio between the number of successful folding runs and the total number of attempted runs, decreases from 100% (in the bulk setup) to 93% if the knotted protein is tethered by the N-terminus, and decreases sharply to 45% when it is surface tethered by the C-terminus. Strictly speaking a direct comparison of the folding rates exhibited by the same protein in the bulk and in the surface tethered setups is not correct because the impediment to move the first (or last) bead is equivalent to change the MC move set by eliminating one of the end-moves. However, it is possible to compare the folding rates of the surface-tethered knotted and unknotted proteins. When knotted protein K is linked to the plane by the N-terminus it folds 1.2 orders of magnitude slower than the unknotted fold in the same folding setup. If, on the other hand, tethering occurs via the C-terminus, the knotted fold achieves the native structure 1.6 orders of magnitude slower than its unknotted counterpart. When the proteins are allowed to fold freely in the bulk, there is only a small difference between their folding rates. In this case, protein U folds 1.5 times faster than protein K. This last observation is in line with very recent results that investigated the folding kinetics of knotted protein (2ouf.pdb) [19] and its designed unknotted counterpart with a similar native fold [49].

We recall that since these proteins have the same gross topology (the absolute *CO* [50] is 17.2 and 16 for protein K and protein U, respectively) the reported differences must mainly reflect the existence of a knot in protein K’s backbone. In particular, surface-tethering the knotted protein at the C-terminus critically impairs folding performance. This observation suggests that the intermediate state that accumulates at *Q* = 0.75 is structurally different from that accumulating when the protein is surface-tethered by the N-terminus (which also hampers folding but to a significantly less extent).

In order to evaluate the importance of the physical constraint imposed by the nearby plane in the surface tethered setup we have conducted a control experiment in which the protein is tethered to a point in space (without the presence of a surface). In this case the ‘foldicity’ attains 100% in the N-tethered setup and 93% in the C-tethered setup. The folding rate, on the other hand, remains considerably low (Figure S1). Taken together these observations confirm that the steric effect imposed by a nearby surface strongly hinders folding efficiency (e.g., by enhancing the formation of topological bottlenecks) and further show that the mobility of the C-terminus is crucial for achieving effective and fast folding of the knotted protein (presumably because the knotting mechanism is based on threading the C-terminus through a knotting loop).

### Influence of Tethering upon Knotting Efficiency

**Figure 6:**
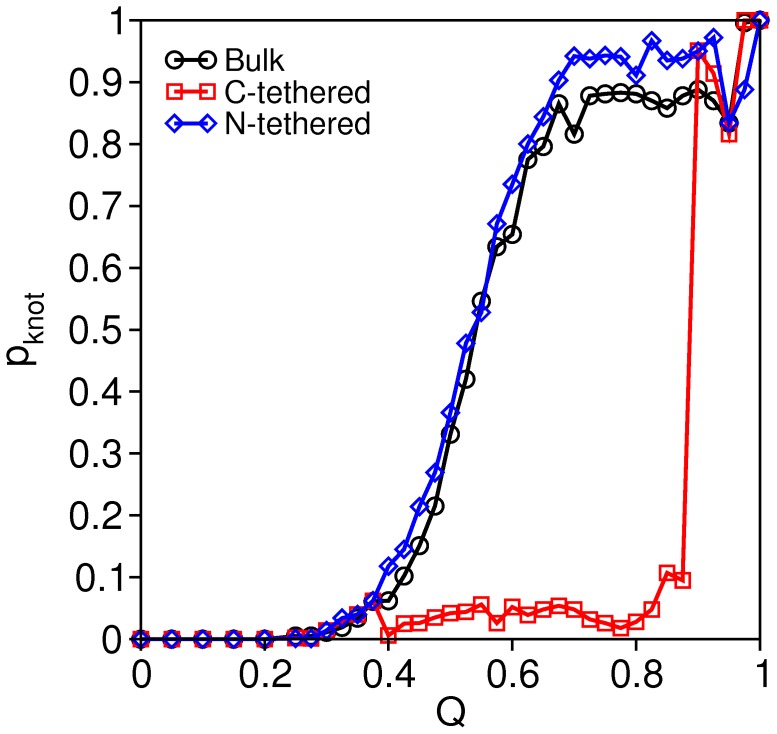
The probability to have the knot formed, p_knot_, as a function of the fraction of established native contacts *Q* in protein K at *T_m_* in the different folding setups.

In this section we investigate the probability of knot formation as protein K gets progressively more native-like, i.e., as the fraction of established native contacts *Q* increases. To carry out this measurement we have constructed an ensemble of 2000 (uncorrelated) conformations for each fraction of native contacts *Q* that were collected from many independent folding trajectories. The KMT algorithm was applied to each conformation to investigate the presence of the knot. The results reported in Figure 6 are strikingly illuminating and they reveal three important points: i) the knotting probability curves display a qualitatively similar sigmoidal shape for both the bulk and surface-tethered (at the N-terminus) folding setups. Specifically, when *Q<*0.4 the probability to have the knot formed is very low (*p*<0.1), increasing up to *p*∼0.9 when *Q>*0.7, i.e., towards late folding, ii) the probability of knot formation is higher when the chain is tethered by the N-terminus. This is particularly clear when *Q*>0.7 and it indicates that fixing the N-terminus enhances knotting efficiency in native-like conformations, iii) when the chain is surface tethered by the C-terminus the probability of knot formation stays very small (*p*<0.1) up to *Q*∼0.8, and it increases sharply to *p*∼0.9 only when *Q*>0.8.

These results show that knotting is typically a late folding event, occurring with highest probability in native-like conformations. They also indicate that the intermediate state (*Q* = 0.75) populated by the protein when it is tethered by the C-terminus (the one that is closest to the knotted core) is structurally different from that forming when tethering occurs at the N-terminus. In particular, the intermediate forming in the N-tethered setup will most likely be knotted, while that forming in the C-tethered setup will most likely be unknotted. To escape these unknotted intermediates it is thus necessary to unfold and refold back in order to get properly tangled, a process that will be hindered not only by the severe steric constraints inside the compact conformation, but also by those imposed by the presence of a nearby plane. Taken together with the results reported in the previous section for the ‘foldicity’ and folding rate the results reported here show that the intermediate states represent topological bottlenecks for folding.

In order to gain further insights into the structural changes occurring towards late folding in the surface-tethered setups we have looked into the dependence of the number of non-native contacts on the fraction of established native contacts, *Q*. We have done so by extracting the relevant data from the set of folding trajectories that were used to evaluate the folding rate. We stress that since protein energetics is modeled by the Gō potential the formation of non-native contacts is strictly geometrically driven and they do not play an energetically stabilizing role.

A comparison between the curves of the bulk folding and the surface tethered folding scenarios reveal an unexpected increase in the number of non-native contacts as a result of surface-tethering which starts at *Q* = 0.75 (and extends over *Q* = 0.9) (Figure S2); this is precisely the fraction of native contacts that corresponds to the minima of the intermediate basin in the free energy profiles (Figure 4). The appearance of this bump in the curves indicates that surface-tethered proteins undergo significant structural re-arrangement in highly native-like conformations. We note that the curve corresponding to the bulk setup is very similar to curves reported in previous lattice investigations based on Gō- and sequence-specific potentials [28], [51].

### Structure of the Intermediate States

In order to isolate and further structurally characterize the intermediate states populated by the knotted protein in the surface-tethered setups we have performed hierarchical clustering (based on contact-map similarity) over an ensemble of 2000 uncorrelated conformations (with *Q* = 0.75) that were collected from many independent folding runs.

**Figure 7:**
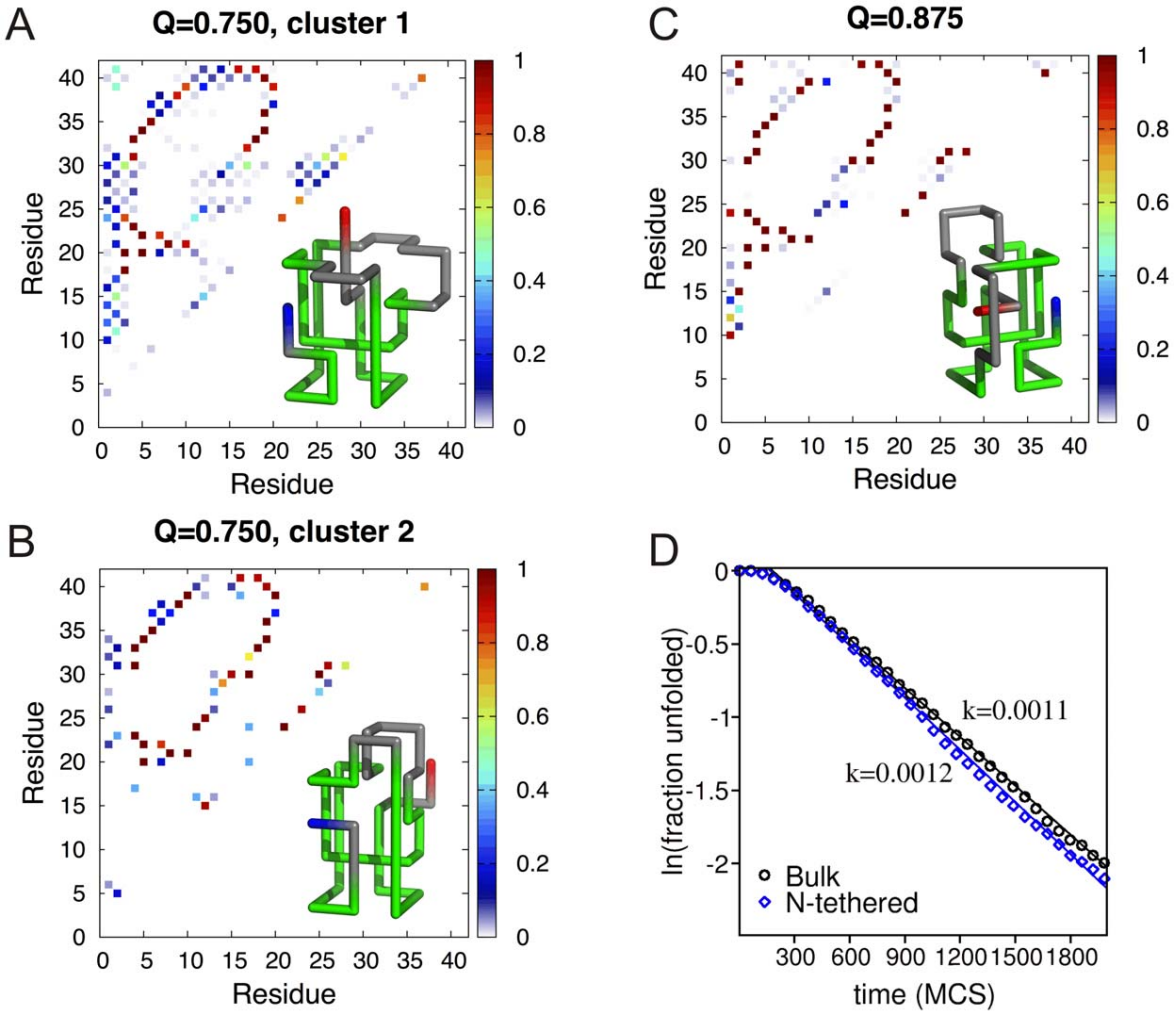
Probability maps showing the probability of occurrence of each established contact (native and non-native) in the two clusters of conformations with fraction of native contacts *Q* = 0.75, which are populated by the knotted protein when it is surface-tethered at the C-terminus. Cluster 1 (A) is the dominant cluster and contains 58% of conformations in the original ensemble while cluster 2 (B) is the sub-dominant cluster containing the remaining 42%. Also shown (inset) is the representative conformation of each cluster. For *Q* = 0.875 we could identify only one ensemble of conformations which is structurally well resolved and represents a folding bottleneck developing from the dominant cluster (C). The green color in the representative conformations highlights the portion of the backbone that is conserved across the three structures. Upon release from the plane and upon surface-tethering at the N-terminus the conformation with *Q* = 0.875 folds rapidly to the native state (D).

We found two relevant conformational clusters when the protein is tethered at the C-terminus, and for each cluster we have evaluated the probability maps for *all* the contacts (native and non-native) established in the analyzed conformations (Figure 7). The larger cluster, that we shall term dominant cluster, contains 58% of all the conformations in the starting ensemble, and the corresponding probability map reveals a structurally blurred state with a strong dominance of non-native contacts establishing between the C-terminus and a long stretch of residues starting at residue 10 and extending to residue 35 (Figure 7). The sub-dominant cluster, containing the remaining conformations, is shown in Figure 7B. The dominant cluster is associated with a non-productive folding pathway leading to a structurally well-resolved ensemble of conformations (with *Q* = 0.875) that represent dead-ends for folding, i.e., it is not possible to achieve the native structure from these conformations (Figure 7C). However, if we start a bulk folding simulation from the representative conformation with *Q* = 0.875 we observe that it rapidly reaches the native fold, the same being true when the simulation is performed with the chain tethered at the N-terminus (Figure 7D). This indicates that the formation of these conformations is strongly rooted on steric effects resulting from the presence a surface near the C-terminus.

A rather different clustering scenario is observed for the knotted protein surface-tethered at the N terminus. In this case the starting ensemble of 2000 conformations with *Q* = 0.75 separates into a major cluster with 90% of the conformations and a minor cluster with the remaining 10%. The probability map for the dominant cluster indicates that it represents a well-resolved conformational state with a few non-native contacts forming with negligible probability (Figure S3). The dominant cluster in this case is therefore associated with a productive folding pathway.

### Insight into the Knotting Mechanism from Structural Clustering

In order to get mechanistic insights into the knotting processes at play in the different folding setups we extended the clustering analysis procedure to ensembles of conformations with fraction of native contacts *Q* and we have extracted the representative conformation of each cluster’s centroid. We recall that the representative conformation is the one that is closest to the average contact map. As in the clustering analysis reported in the previous section, we considered starting ensembles of 2000 uncorrelated conformations. We have started with *Q* = 0.3 (corresponding to 12 native contacts) because it is highly unlikely to observe knot formation in conformations with smaller *Q* (we recall that the number of native contacts corresponding to the knotted core is 8). We have thus obtained a succession of conformations of increasing *Q* that provides insight into the structural changes underlying the knotting mechanism at play in each set-up. At this point it is important to recall that structural similarity between two conformations (as measured by *Q*) does not necessarily imply that they are kinetically close (i.e. that they can interconvert easily into one another) [34]. Therefore, it is possible that large free energy barriers exist between successive *Q* values. For this reason we kept only the representative conformations that can convert into each other without significant structural rearrangement.

**Figure 8:**
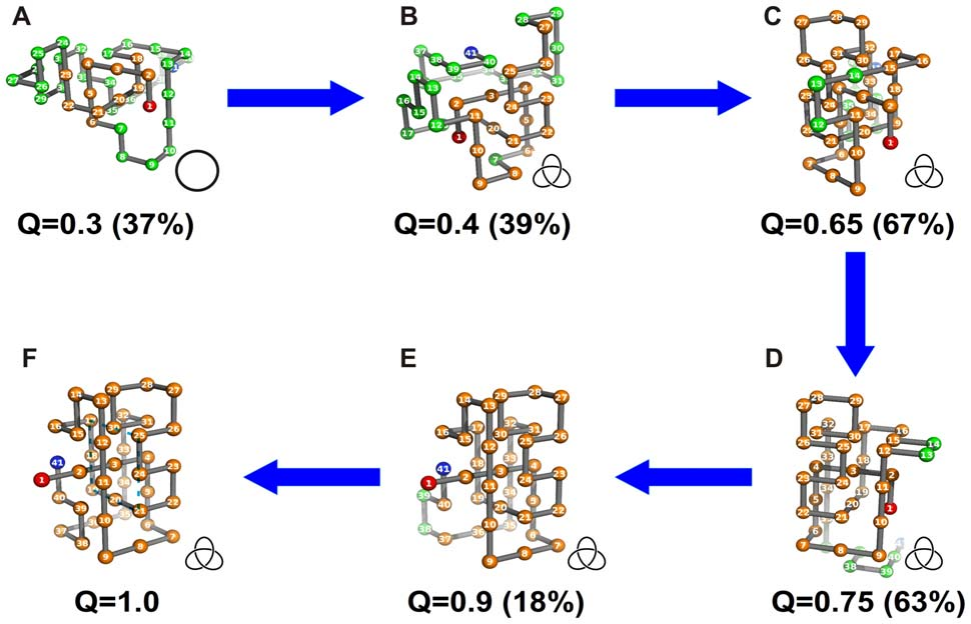
Knotting mechanism operating in the bulk. Each conformation with fraction of native contacts *Q* is the closest to the cluster’s centroid, and is taken as the cluster’s representative. The residues colored in orange have at least two of its native contacts formed. In parenthesis we show the ratio between the size of the cluster (i.e. its number of conformations) and the size of the initial ensemble of conformations with fraction of native contacts *Q*. In (D) we highlight the loop formed by residues 17–21, 24,25,30.

In Figure 8 we report a knotting mechanism that is likely to be observed in the bulk set-up. When occurring in the bulk, the knotting mechanism is based on a threading movement of the terminal bead that is closest to the knotted core (i.e. the C-terminus). This movement can either occur through a loosely formed loop in conformations with a small fraction of native contacts established (Figure 8A) - and in this case the threading movement does not necessarily knot the fold- or through a tightened loop formed in conformations which are more native-like (Figure S4D), which effectively knots the fold. Eventually this loop develops into the native loop formed by residues 17–21, 24, 25, 30 (Figure 8F).

**Figure 9:**
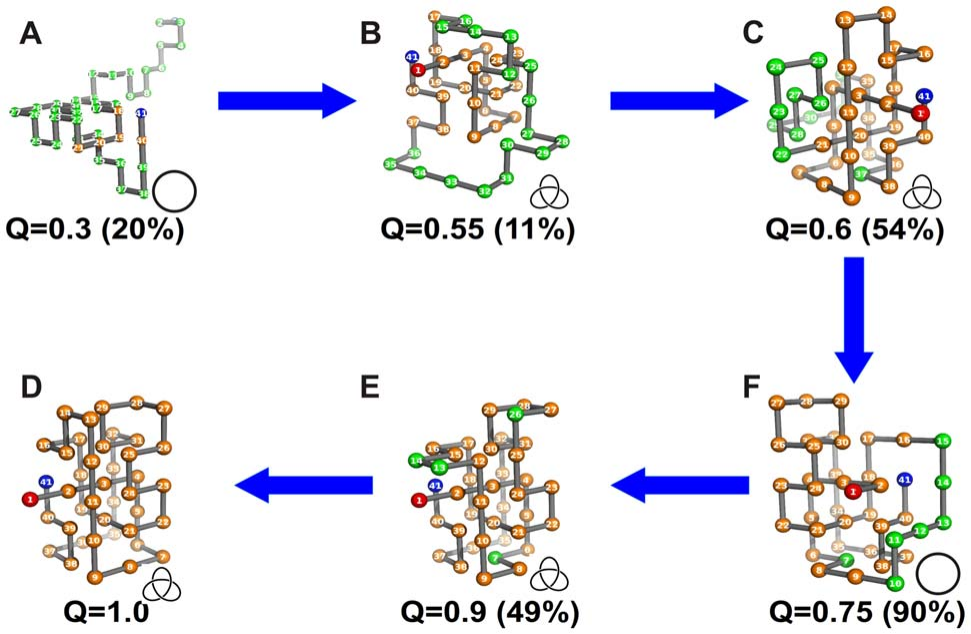
Knotting mechanism operating when the chain is surface tethered by the N- terminus.

The late threading of the C-terminus is actually the observed mechanism when the N-terminus is surface tethered (Figure 9). In this case, in order to observe productive folding it is important to keep in its native position the terminal segment formed by beads 37–41 by establishing native contacts with beads 18–20 (Figure 9B). If this structural restraint is not imposed from early on in folding there is the possibility to form highly native-like conformations, which, despite being knotted, will not find the native structure if they are kept surface-tethered (Figure S5).

**Figure 10:**
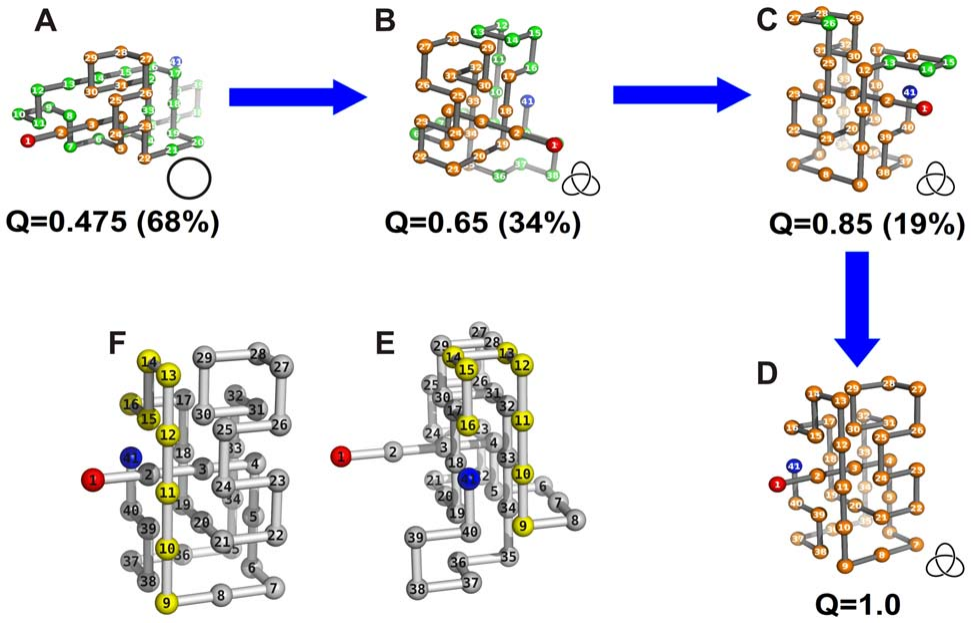
Knotting mechanism operating when the chain is surface tethered by the C-terminus. The conformations in the inner panel are the representative conformations with *Q* = 0.65 (E) and native structure (F) where the chain segment 9–16 is highlighted. Tethering the protein at the C-terminus forces this segment to develop in a plane clearly behind the native plane.

If the chain is linked to the plane by its C-terminus there is a completely different knotting mechanism, which we term the spindle mechanism (Figure 10). In order to observe successful folding in this setup it is necessary that the first four beads of the protein form a line segment that remains formed throughout the folding process (Figure 10A). This segment acts like a spindle around which the chain enlaces itself. The formation of the spindle appears to be a necessary condition for successful folding. If it does not form there is a misfolding process that terminates in dead-end conformations (Figure S6 and Figure S7). Contrary to what is observed in the N-tethered setup, these misfolded conformations are not knotted and therefore require substantial structural re-arrangement in order to fold properly once they are released from the plane (Figure S6F and Figure S7F).

Overall the results reported here show that the presence of a nearby plane makes the folding process of the C-tethered chain particularly prone to error (i.e. misfolding). It is interesting to note that even when successful folding is observed in this setup the nearby plane still hampers the formation of the native structure. In particular, the protein’s surface that is closest to the C-terminus (highlighted in yellow in Figure 9F) is forced to develop behind its ‘native plane’ (Figure 9E).

### Conclusions

In this work we have explored the consequences on the folding process of tethering a lattice protein to a chemically inert plane. The native state of this lattice protein is tangled into a trefoil knot. The minimal chain segment that contains the knotted core is closer to one terminus, which we named as C-terminus (by analogy with the location of the knotted core in real trefoil proteins).

The first conclusion from this study is that the outcome of the tethering experiment depends critically on which terminus is used to link the protein to the plane. In particular, if it occurs through the C-terminus, the folding process becomes severely hampered. The lack of folding efficiency shows itself through a low folding rate. Actually, the process becomes so incredibly slow that folding to the native structure could not be observed in all the attempted folding trajectories. The reason is that the chain gets trapped into a post transition state intermediate that is a topological bottleneck (i.e. a compact native-like conformation, which is not knotted). The formation of these conformations is strongly rooted on steric interactions with the plane. The latter are also important (but not to the same extent) when the chain is surface- tethered at the N-terminus, the one that is placed more far away from the knotted core. Indeed, although we could also observe the formation of topological bottlenecks in this setup, these structures are knotted and can fold easily once released from the plane. It is interesting to note that steric interactions with the plane affect more the folding process of the knotted protein than its unknotted counterpart suggesting that this kind of interactions may be more relevant for this class of proteins.

We have interpreted the above observations in terms of knotting mechanisms. Our investigation highlights the importance of a knotting mechanism based on a threading movement of the C-terminus (the one closest to the knotted core) through a loop. This mechanism operates both in the bulk setup and when the protein is tethered at the N-terminus. If the protein is tethered to the plane by the C-terminus the threading movement is blocked. However, the chain is still able to find an alternative way to get tangled via the so-called spindle mechanism. In the spindle mechanism the first 4 residues of the C-terminus must arrange themselves into a line segment that acts like a spindle around which the chain twines itself. Although this mechanism is capable of folding the chain, it is considerably less efficient than the threading mechanism, in part due to the stringent structural constraint imposed on the terminus’ beads. The spindle mechanism is a prediction of our lattice model simulations and, to the best of our knowledge, it was not yet observed in other simulations or *in vitro* experiments.

A common feature of the folding process in the three setups is that knotting is most likely to occur towards late folding in conformations that are substantially structurally consolidated. This observation is in line with our previous simulation studies [21] where we measured the knotting probability as a function of the folding probability *p_fold_* reaction coordinate, with simulation studies from other groups [10], [13], and also in agreement with recent experimental data [18].

Since tethering the protein at the C-terminus also occurs in co-*translational* folding it is interesting to reflect upon the consequences of the results reported here for co-translational folding of knotted proteins.

A series of very interesting results on co-translational folding of (unknotted) proteins has been reported recently [52], [53], [54]. A major conclusion from these studies is that the co-translational folding process of topologically complex proteins differs significantly from that observed in the bulk [53]. Indeed, not only these proteins tend to populate intermediate states while they are being synthesized (in sharp contrast with the high cooperativity characteristic of their bulk folding transition), as they also tend to fold considerably slower. Since knotted proteins represent extreme examples of topologically complex folds we may anticipate, based on the results for unknotted proteins that their co-translational folding process will differ sharply from that observed in the bulk. Although we have looked into full-length chains (i.e. completely synthesized proteins) tethered to a plane, our results are in line with those of O’Brien and co-workers. We also observe formation of intermediate states, and a very low folding rate upon tethering. Moreover, since the knotted core of proteins with trefoil knots is closer (or indeed very close) to the C-terminus, which is tethered to the ribosome, the knotted core will only be complete when protein synthesis itself is near completion (we recall that in protein synthesis the chain grows from the N- to the C-terminus). Thus, while it is possible to form native structure during co-translational folding, correct knotting (and, by extension, productive folding) will only be possible for nearly synthesized chains. Finally, since the protein is tethered to the ribosome by the C-terminus during protein synthesis, a co-translational folding mechanism of trefoils based on threading the C-terminus through a loop is not possible. The arrested growing chains will have to start folding by their N-terminus, following an alternative mechanism. Our results suggest that folding mechanisms that are not based on loop threading may lack efficiency. Taken together these observations thus suggest that co-translational folding of knotted trefoils will be highly impaired relative to the bulk process and possibly need to be assisted by chaperones.

We hope that the present study will inspire future experimental work focused on the folding of knotted proteins. The results presented here suggest that the use of single molecule experiments can be particularly useful to reveal the preferred folding mechanisms operating on knotted trefoil proteins and disclosing alternative ways to tangle the protein backbone.

## Supporting Information

Figure S1 Comparison of the folding rates in the surface-tethered setups and in the point-tethered setups.
(TIFF)

Figure S2 Mean number of established non-native contacts as a function of the fraction of established native contacts, *Q*. (A) for the knotted protein and (B) for the unknotted one. To compute these curves we have extracted the relevant data from the set of folding trajectories that were used to evaluate the folding rate (Figure 5). Essentially, we grouped the conformations sampled by the protein in the 2000 MC runs according to their fraction of native contacts, and computed the mean averaged number of non-native contacts in each conformational ensemble. The bump developing from *Q*∼0.75–0.9 indicates that surface-tethered proteins undergo structural re-arrangement in highly compact, native-like conformations. These conformational excursions occur both in the knotted and unknotted folds, although they appear more significant in the case of the knotted fold. Indeed, the plot for protein U is noisy in the *Q* range of interest and the number of established non-native contacts is also smaller in that case.
(TIFF)

Figure S3 Probability map in the dominant cluster of conformations with fraction of native contacts *Q = *0.75 populated by the knotted protein when it is linked to the plane by its N-terminus. The probability map shows the mean averaged probability of occurrence of each established contact (native and non-native). Also shown (inset) is cluster’s representative conformation that is knotted. The part of the backbone highlighted in green is the knotted core.
(TIFF)

Figure S4 Alternative knotting mechanism operating in the bulk. Each conformation with fraction of native contacts *Q* is the closest to the cluster’s centroid, i.e., the cluster’s representative. The residues colored in orange have at least two of its native contacts formed. In parenthesis we show the ratio between the size of the cluster (its number of conformations) and the initial ensemble from which they were clustered where conformations have fraction of native contacts *Q*.
(TIF)

Figure S5 Pathway leading to dead-end conformations when the chain is tethered to the surface via the N-terminus. Each conformation with fraction of native contacts *Q* is the closest to the cluster’s centroid, i.e., the cluster’s representative. The residues colored in orange have at least two of its native contacts formed. In parenthesis we show the ratio between the size of the cluster (its number of conformations) and the initial ensemble with fraction of native contacts *Q*.
(TIF)

Figure S6 Pathway leading to dead-end conformations when the chain is tethered to the surface via the C-terminus. Each conformation with fraction of native contacts *Q* is the closest to the cluster’s centroid, i.e., the cluster’s representative. The residues coloured in orange have at least two of its native contacts formed. In parenthesis we show the ratio between the size of the cluster (its number of conformations) and the initial ensemble with fraction of native contacts *Q.*
(TIF)

Figure S7 Another folding pathway leading to dead-end conformations when the chain is tethered to the surface via the C-terminus. Each conformation with fraction of native contacts *Q* is the closest to the cluster’s centroid, i.e., the cluster’s representative. The residues coloured in orange have at least two of its native contacts formed. In parenthesis we show the ratio between the size of the cluster (its number of conformations) and the initial ensemble with fraction of native contacts *Q*.
(TIF)
