# Supplementary figures and images for: How Difficult Is It to Fold a Knotted Protein? In Silico Insights from Surface-Tethered Folding Experiments
Source: PLoS One. 2012 Dec 20;7(12):e52343. doi: 10.1371/journal.pone.0052343 (PMC3527535; doi:10.1371/journal.pone.0052343)

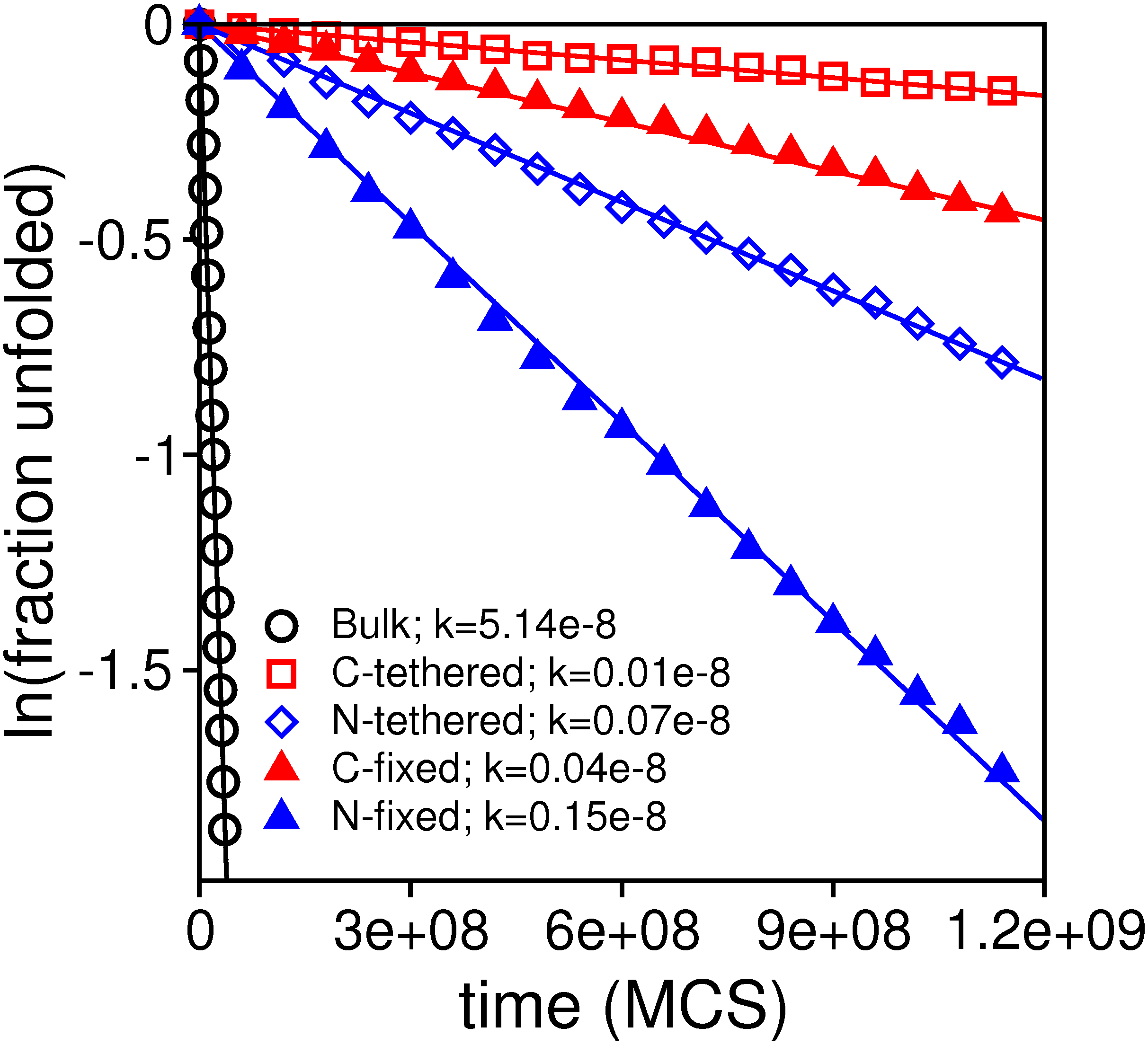

Supplement: Figure S1 — Comparison of the folding rates in the surface-tethered setups and in the point-tethered setups. (TIFF) [file pone.0052343.s001.tiff]

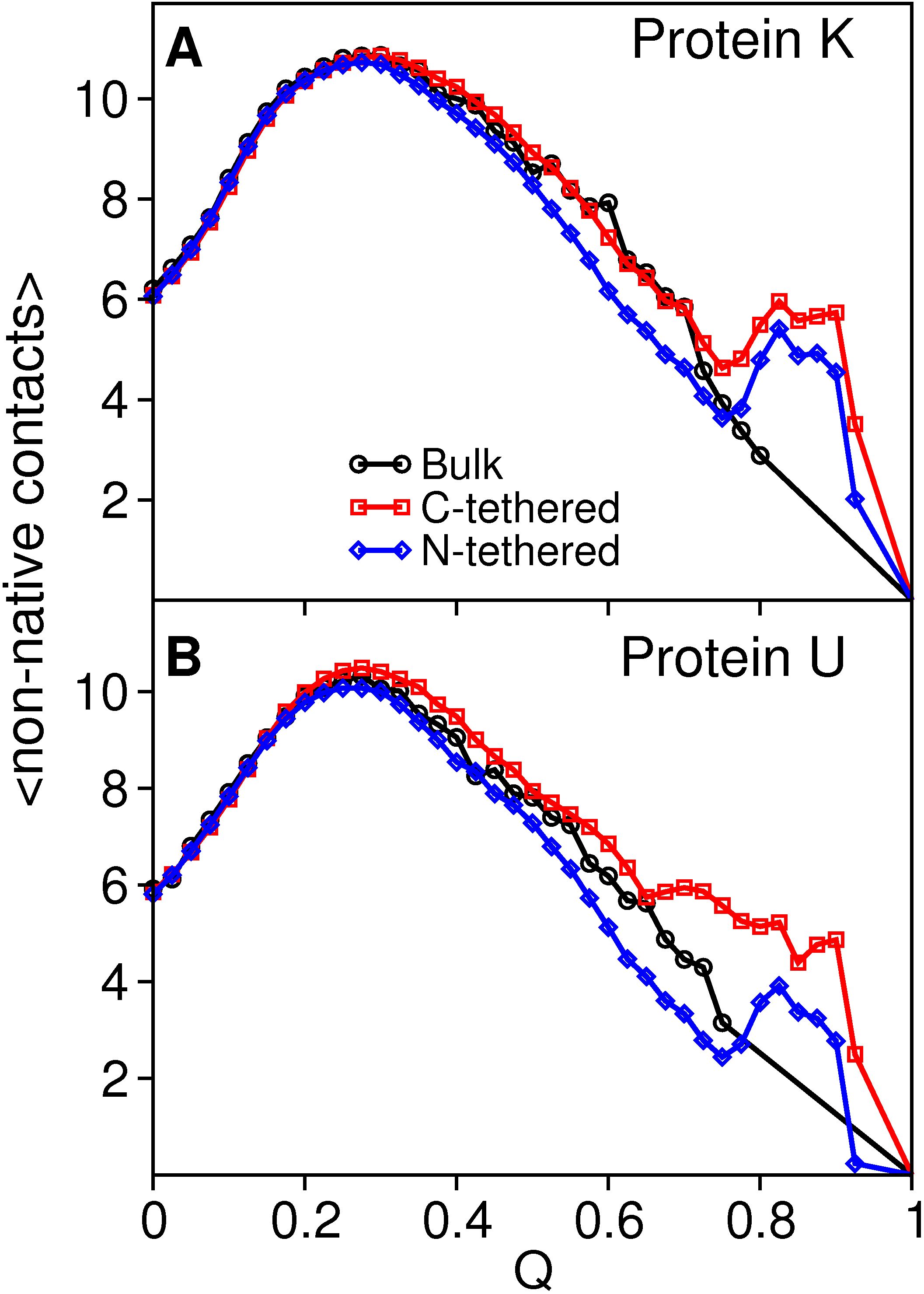

Supplement: Figure S2 — Mean number of established non-native contacts as a function of the fraction of established native contacts, Q. (A) for the knotted protein and (B) for the unknotted one. To compute these curves we have extracted the relevant data from the set of folding trajectories that were used to evaluate the folding rate (Figure 5). Essentially, we grouped the conformations sampled by the protein in the 2000 MC runs according to their fraction of native contacts, and computed the mean averaged number of non-native contacts in each conformational ensemble. The bump developing from Q∼0.75–0.9 indicates that surface-tethered proteins undergo structural re-arrangement in highly compact, native-like conformations. These conformational excursions occur both in the knotted and unknotted folds, although they appear more significant in the case of the knotted fold. Indeed, the plot for protein U is noisy in the Q range of interest and the number of established non-native contacts is also smaller in that case. (TIFF) [file pone.0052343.s002.tiff]

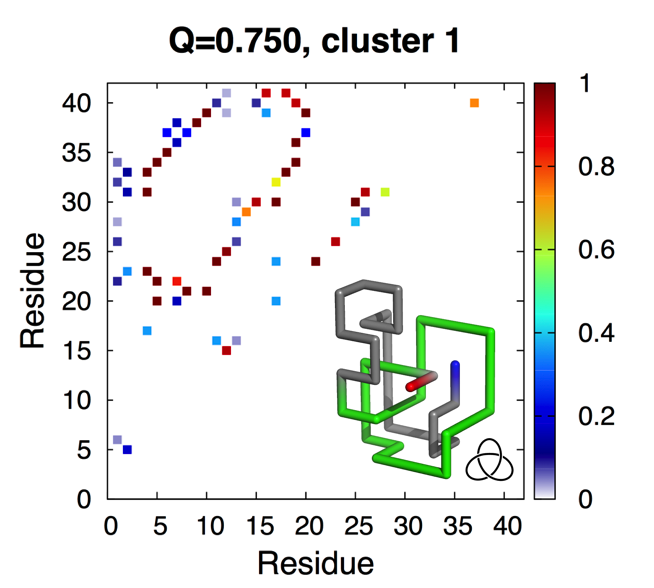

Supplement: Figure S3 — Probability map in the dominant cluster of conformations with fraction of native contacts Q = 0.75 populated by the knotted protein when it is linked to the plane by its N-terminus. The probability map shows the mean averaged probability of occurrence of each established contact (native and non-native). Also shown (inset) is cluster’s representative conformation that is knotted. The part of the backbone highlighted in green is the knotted core. (TIFF) [file pone.0052343.s003.tiff]
